# Largely reduced OAR doses, and planning and delivery times for challenging robotic SBRT cases, obtained with a novel optimizer

**DOI:** 10.1002/acm2.13172

**Published:** 2021-01-21

**Authors:** Marta K. Giżyńska, Linda Rossi, Wilhelm den Toom, Maaike T. W. Milder, Kim C. de Vries, Joost Nuyttens, Ben J. M. Heijmen

**Affiliations:** ^1^ Erasmus MC Cancer Institute University Medical Center Rotterdam Department of Radiotherapy Dr.Molewaterplein 40 Rotterdam 3015 GD The Netherlands

**Keywords:** CyberKnife SBRT, sequential optimizer, treatment planning for prostate and lung, VOLO™

## Abstract

Recently, VOLO™ was introduced as a new optimizer for CyberKnife**®** planning. In this study, we investigated possibilities to improve treatment plans for MLC‐based prostate SBRT with enhanced peripheral zone dose while sparing the urethra, and central lung tumors, compared to existing Sequential Optimization (SO). The primary focus was on reducing OAR doses. For 25 prostate and 25 lung patients treated with SO plans, replanning with VOLO™ was performed with the same planning constraints. For equal PTV coverage, almost all OAR plan parameters were improved with VOLO™. For prostate patients, mean rectum and bladder doses were reduced by 34.2% (*P* < 0.001) and 23.5% (*P* < 0.001), with reductions in D_0.03cc_ of 3.9%, 11.0% and 3.1% for rectum, mucosa and bladder (all *P* ≤ 0.01). Urethra D_5%_ and D_10%_ were 3.8% and 3.0% lower (*P* ≤ 0.002). For lung patients, esophagus, main bronchus, trachea, and spinal cord D_0.03cc_ was reduced by 18.9%, 11.1%, 16.1%, and 13.2%, respectively (all *P* ≤ 0.01). Apart from the dosimetric advantages of VOLO™ planning, average reductions in MU, numbers of beams and nodes for prostate/lung were 48.7/32.8%, 26.5/7.9% and 13.4/7.9%, respectively (*P* ≤ 0.003). VOLO™ also resulted in reduced delivery times with mean/max reductions of: 27/43% (prostate) and 15/41% (lung), *P*  < 0.001. Planning times reduced from 6 h to 1.1 h and from 3 h to 1.7 h for prostate and lung, respectively. The new VOLO™ planning was highly superior to SO planning in terms of dosimetric plan quality, and planning and delivery times.

## 1 Introduction

Recently, the novel VOLO™ inverse treatment planning optimizer for SBRT planning for the CyberKnife® System (CK) was implemented in the Precision® treatment planning system (Accuray Inc, Sunnyvale, USA). VOLO™ has major differences in optimization approach compared to the existing Sequential Optimization (SO). Three recent studies showed increased plan efficiency for VOLO™^1^, ^2^, ^3^ compared to SO. All three studies were performed with a limited number of patients per tumor site: five to ten. Schüler et al.^1^ compared the VOLO™ and SO algorithms for five prostate cancer patients for treatment with the InCise™ MLC. The study by Calusi et al.^2^ included seven prostate cancer patients planned with MLC. In both studies, five fractions of 7.25 Gy were delivered, without explicit intra‐prostatic dose shaping to spare the urethra. For lung tumors (location not specified), a VOLO™‐SO comparison was only made for the IRIS™ variable aperture collimator.^3^ As it was hypothesized that VOLO™ might provide better dosimetric results for complex SBRT treatments,^2^ we decided to compare VOLO™ with SO for twenty‐five prostate cancer patients with complex intra‐tumor dose prescription, and 25 central lung cancer patients. All plans were made for treatment with the InCise™ 2 MLC. Primary aim was to explore whether VOLO™ planning could be used to further minimize OAR doses, aside from possible efficiency improvements, e.g., regarding delivery times and MU. Compared to,^1^, ^2^ our prostate plans had an enhanced complexity as brachytherapy‐like dose distributions were generated, with enhanced dose in the peripheral zone, while selectively avoiding the highest doses in the urethra.^4^, ^5^ With higher dose per fraction (4 × 9.5 Gy), our planning protocol was more challenging, as also lower dose‐volume constraints for rectum and bladder were required. For lung cancer, the new VOLO optimizer was challenged with central tumors, where OARs are closer to the target making the treatment protocol more difficult to be fulfilled than in peripheral tumors.

## 2 Methods

### 2.A Treatment unit

In this study, plans were prepared for CyberKnife® M6 system, as used in our center and equipped with three types of collimation: fixed cones, IRIS™ collimator (both producing circular fields of diameter between 5 and 60 mm) and the InCise™ 2 MLC.^6^, ^7^, ^8^, ^9^, ^10^, ^11^ The latter can produce a maximum field size of 11.5 cm × 10 cm using 26 pairs of leafs of 3.85 mm width @ SAD 800 mm. In this study, all SO and VOLO plans were optimized for treatment with the MLC.

### 2.B Optimization algorithms and application

#### 2.B.1 Sequential optimization (SO)

SO has been described in detail in the literature.^6^, ^12^, ^13^ Here, a short summary is provided. In SO, MLC segments are generated based on Beam’s‐Eye‐View projections (conformal with or without OAR cutouts; eroded, perimeter and random shapes are allowed). Next, the segment weights are optimized in a stepwise optimization approach utilizing linear programming. The user can define hard planning constraints and objectives. Each objective is individually optimized, in order of attributed priority, without violating imposed constraints. After each objective optimization, an extra constraint is added to the optimization problem; the involved objective is transformed into a constraint with a slightly relaxed attained objective value. After initial optimization, a node and segment reduction and reoptimization iterative procedure can be applied to limit treatment time. The latter was done for all SO plans used in this study. The optimizations run on a CPU.

#### 2.B.2 VOLO™

Plan optimization with VOLO™ is based on a weighted‐sum cost function; there are no hard constraints.^1^, ^2^, ^3^, ^6^ This cost function combines multiple dose‐volume terms, with corresponding user‐defined weighting factors. In a first phase, optimal fluence maps are generated while including a fluence smoothness term in the cost function, with a user‐defined weighting factor. A variant of the quasi Newton algorithm, L‐BFGS‐B, is used as optimizer.^14^ Each fluence map is then segmented to generate a set of initial MLC apertures that comply with the physical limitations of the MLC. Segment optimization follows the fluence optimization and is divided into three phases. In preadaptation, the fluence optimization result is segmented and dose is recalculated for each defined segment and then the segment weights are reoptimized.^15^ The cost function is the same as that used during fluence optimization except that the smoothness penalty is replaced by a total MU penalty. In the preadaptation phase, the segment weight optimization is performed iteratively with low MU segments removed at each iteration. Subsequently, leaf position adaptation is performed to fine tune the apertures.^16^ Finally, in the post adaptation phase, dose is recalculated for the adjusted segments and segment weights are reoptimized together with iterative pruning of low MU segments. VOLO™ optimization is implemented on a GPU. It should be noted that both SO and VOLO™ provide an option to select a randomized and spatially distributed subset of nodes prior to optimization. This was not used for any of the plans in this study. Instead for both SO and VOLO™, optimization started with all possible nodes available in the selected robot motion path.

#### 2.B.3 Treatment planning

Both for prostate and lung, SO was used for generation of the clinical plans that were used in this study for comparison with VOLO™. The SO plans were made by expert CK planners, acknowledging the planning complexity and the need for high‐quality plans because of the delivered high fraction and total doses. The VOLO™ plans were generated by a postdoctoral researcher/medical physicist (MG) who started the project without prior experience in CK or SBRT planning. This VOLO™ planner first received a brief introduction to the system by an Accuray representative. Next, a similar training procedure was applied for both tumor sites: after discussions with a CK planner (WT) and treating clinicians (KdV or JN), plans were generated for five arbitrarily selected training patients. In this training phase, the planner had access to the SO plans of the training patients, and plans were iteratively improved by feedback of the expert planner or clinicians. After the training phase, the postdoc generated the plans for the other twenty study patients, without having knowledge of the clinical dose distributions and without feedback by the expert planner or clinicians.

#### 2.B.4 Plan evaluation and comparison

Both for prostate and lung, VOLO™ plans for ten arbitrarily selected patients were evaluated by a clinician to decide whether the plans were acceptable for clinical use. To this purpose, the plans were shown to the physician in the Precision TPS who examined the dose distribution in CT slices and DVHs considering protocol constraints. In this procedure, the clinicians had no access to the clinically delivered SO plan. VOLO™ and SO plans were renormalized to exactly meet the clinically requested PTV coverage, followed by comparisons of dosimetric and nondosimetric plan parameters (MU, estimated treatment time, and number of nodes, beams and segments), see details for both tumor sites below. Conformity Index (CI) was calculated as volume receiving the prescribed dose divided by PTV. Near‐maximum dose, D_0.03cc_, was reported as a surrogate of D_max_.^17^ To assess plan complexity, the average weighted segment size (WSS) and the Modulation Complexity Score (MCS,^18^) were calculated. A plan can achieve MCS values from 0 (the highest complexity) to 1 (the lowest complexity). For VOLO™, planning times were measured, while for the clinical SO planning the planning times were estimated by planners.

### 2.C Prostate

#### 2.C.1 Patients and planning protocol

The twenty‐five arbitrarily selected prostate patients included in the study were treated in our clinic between November 2016 and August 2019. Total dose was delivered in four daily fractions of 9.5 Gy (38 Gy total dose). PTV dose distributions were intentionally heterogeneous with enhanced dose in the peripheral zone while restricting the urethra dose.^4^, ^5^ The PTV coverage goal was 95%, with an imposed maximum dose of 62.5 Gy. The intention was to limit D_max_ for rectum to 38 Gy, for rectal mucosa to 28.5 Gy, and for bladder to 41.8 Gy, and to keep near‐maximum doses, D_1cc_, in rectum and bladder below 32.3 Gy and 38 Gy, respectively. When considered unfeasible, the 1 cc could be enlarged to 1.2 cc and 1.5 cc, respectively. The goal was to keep urethra D_50%_, D_10%_ and D_5%_ below 40 Gy, 42 Gy and 45.5 Gy, respectively. Femoral heads should receive less than 24 Gy. No beams were allowed to pass through the penis and scrotum.

#### 2.C.2 SO and VOLO™ planning

All clinical plans were generated with SO as implemented in TPSs supplied by Accuray Inc; seven with MultiPlan 5.3.0, and eighteen with Precision 1.1.1.1. In both TPSs, the implementation of SO was the same. All VOLO™ plans were generated in Precision 2.0.0.0. The same dose calculation algorithm, i.e., Finite Size Pencil Beam (FSPB), treatment machine (above) and prostate node path (full or short as decided during clinical planning) were used for SO and VOLO™. In order to create VOLO™ plans with a similar PTV dose as SO, the average mean PTV dose in the 25 SO plans was used for guidance in the VOLO™ planning. For 12 out of 25 SO plans, an additional blocking structure was used to avoid beams going through the belly. This structure was omitted in VOLO™ planning. In SO plans, heterogeneous dose distributions were obtained by dividing the PTV into a peripheral (outer 5mm) and an inner zone (without urethra), and using different planning objectives for them. To obtain inhomogeneous PTV doses with VOLO™ planning, the PTV with subtracted OARs was added as an extra planning structure with objectives for dose enhancements: V_60%_ ≥ 48Gy and V_25%_ ≥ 55Gy (with possible small patient‐specific variation). Urethra high dose was controlled by using dedicated constraints (SO) or objectives (VOLO™). According to general clinical practice, if achieving desired 95% PTV coverage and fulfilling OARs constraints as specified in planning protocol was not possible at the same time, PTV coverage was reduced until OARs constraints were met.

#### 2.C.3. Plan evaluations and comparisons

Prior to the pairwise dosimetric comparisons of SO and VOLO™ plans, all plans were normalized to have the clinically requested PTV coverage of 95%. Estimated delivery times were calculated for 120 sec imaging intervals. Patient setup time was not included.

### 2.D Lung

#### 2.D.1 Patients and planning protocol

Twenty‐five central lung cancer patients treated in our clinic between March 2019 and January 2020 were replanned with VOLO™. Patients were treated with five daily fractions of 11 Gy. The PTV was aimed to have a coverage of 98% with a maximum dose between 69 Gy and 78.57 Gy. The intention was to limit D_max_ in the spinal cord to 27 Gy, in the trachea to 45 Gy, in the main bronchus to 50 Gy, in the plexus brachialis to 30 Gy, and in the esophagus, stomach, bowel and skin to 35 Gy. When 30 Gy was considered infeasible, 40 Gy could be accepted as D_max_ in esophagus. The goal was to keep lung V_16Gy_ below 31 %, and thoracic wall V_30Gy_ below 30 cc.

#### 2.D.2 SO and VOLO™ planning

Nineteen clinical plans were generated with Precision 1.1.1.1, and six with Precision 2.0.1.1, with both systems having the same SO implementation. All VOLO™ plans were generated in Precision 2.0.1.1. The same treatment machine (above), nodes path (full or short as decided during clinical planning) and blocking structures were used for planning in SO and VOLO™. For SO, a first optimization was done with FSPB with Lateral Scattering (FSPB + LS) and after achieving satisfactory results optimization with the Monte Carlo dose calculation engine (MC) was performed with parameters found previously. With VOLO™, FSPB is used during fluence optimization, FSPB + LS in the preadaptation phase of segment optimization, while MC was used in the postadaptation phase. For final dose calculations in VOLO™, MC was used for the same requested uncertainty as used for SO. In order to create VOLO™ plans with similar PTV doses as obtained with SO, maximum SO PTV doses were used to guide the VOLO™ planning. To overcome the lack of MC in the VOLO™ fluence optimization step and therefore lack of dose in low‐density regions of PTV surrounding GTV, the additional structure called PTV‐ring (PTV minus GTV minus OARs) was used with higher minimum dose set as objective for all VOLO™ plans. According to general clinical practice, if achieving desired 98% PTV coverage and fulfilling OARs constraints as specified in planning protocol was not possible at the same time, PTV coverage was reduced until OARs constraints were met, similar as for prostate cancer (above).

#### 2.D.3 Plan evaluations and comparisons

Prior to the pairwise comparisons of SO and VOLO™ plans, all plans were normalized to have the same, clinically requested PTV coverage of 98%. Delivery time calculations were made for 60 sec imaging intervals. Patient setup time was not included.

### 2.E Statistics

Two‐sided Wilcoxon signed‐rank tests were used for statistical analyses with p‐values lower than 0.05 indicating statistical significance.

## 3 Results

### 3.A Prostate

#### 3.A.1 Clinical acceptability of original VOLO™ plans

For the evaluating clinician (KdV), all plans in the arbitrarily selected subset of 10 patients were clinically acceptable.

#### 3.A.2 PTV coverage and dose

Prior to renormalization, PTV coverages achieved with SO ranged from 86.7% to 98.1%, with in total 15 patients below 95%. Two patients had a coverage below 90%, eight – between 90% and 94%, and five between 94% and 95%. Plans created with VOLO™ had PTV coverages between 89% and 95.1%, with only five patients below 95%. Of those five patients, one had a coverage below 90%, two – 93%, and two between 94.5% and 95%.

For all 15 patients with an SO plan with PTV coverage < 95%, the VOLO™ optimizer could achieve 95% coverage for twelve of them. For the other three patients, VOLO™ increased it from/to 86.7/93.0%, 87.9/89.0%, 93.6/94.5%, while fulfilling all OAR constraints. For patient 21, PTV coverage with VOLO™ was 93% while the SO plan had a coverage of 95%, because of a clinically accepted violation in bladder V_38Gy_, not reproduced in the VOLO™ plan. Tradeoffs for all prostate patients are presented in electronic Appendix S1.

**FIG. 1:**
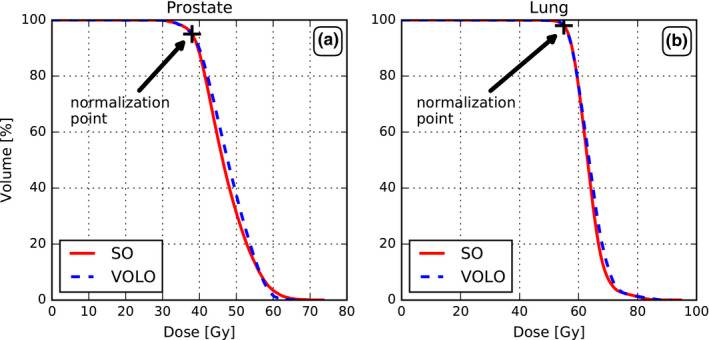
Comparison of SO and VOLO™ in terms of population mean PTV DVHs for prostate (a) and lung (b).

In Fig. 1(a) population mean PTV DVHs after renormalization to 95% are presented, showing large similarity for the two planning approaches. Table 1 also demonstrates that PTV D_98%_, D_0.03cc_, and the CI were similar.

**TABLE 1:** Comparisons of plan parameters for prostate cancer patients for plans generated with SO and VOLO™.

	SO		VOLO™		SO‐VOLO™^a^		*P*‐value^b^
	Mean	Range	Mean	Range	Mean [%]	Range [%]	
PTV							
D_98%_ [Gy]	35.2	[33.4, 37.3]	35.1	[32.6, 36.2]	0.3	[−4.9, 4.7]	0.5
D_0.03cc_ [Gy]	62.2	[54.3, 72.8]	61.3	[59.1, 67.7]	0.9	[−11.4, 16.5]	0.4
CI	1.2	[1.1, 1.4]	1.2	[1.1, 1.3]	−2.1	[−12.2, 11.0]	0.1
Rectum							
D_0.03cc_ [Gy]	37.7	[35.2, 43.0]	36.2	[34.1, 41.6]	3.9	[−3.3, 15.4]	<0.001
D_1cc_ [Gy]	31.6	[27.5, 36.6]	30.0	[26.4, 33.3]	4.7	[−2.3, 20.5]	<0.001
D_mean_ [Gy]	11.6	[9.3, 15.9]	7.6	[5.3, 9.8]	34.3	[20.9, 45.8]	<0.001
V_40GyEq_ [%]	9.5	[4.2, 15.9]	6.0	[3.2, 10.7]	34.5	[−6.1, 66.5]	<0.001
V_60GyEq_ [%]	3.5	[1.5, 6.6]	2.4	[1.1, 4.0]	27.7	[−21.0, 71.9]	<0.001
Rectal Mucosa							
**D_0.03cc_ [Gy]**	29.0	[26.8, 34.0]	25.7	[23.3, 27.6]	**11.0**	[0.0, 31.5]	**<0.001**
Bladder							
D_0.03cc_ [Gy]	42.2	[39.0, 51.7]	40.8	[38.7, 46.9]	**3.1**	[−14.1, 16.9]	**0.01**
D_1cc_ [Gy]	38.7	[34.6, 44.5]	36.9	[34.1, 42.7]	**4.7**	[−7.6, 12.1]	**<0.001**
D_mean_ [Gy]	12.3	[8.1, 17.3]	9.3	[7.1, 12.3]	**23.5**	[−10.0, 41.0]	**<0.001**
Urethra							
D_5%_ [Gy]	43.1	[39.7, 50.9]	41.4	[39.5, 45.4]	**3.8**	[−3.9, 14.9]	**0.001**
D_10%_ [Gy]	42.3	[39.4, 49.9]	41.0	[39.1, 45.1]	**3.0**	[−3.8, 14.0]	**0.002**
D_50%_ [Gy]	40.0	[36.6, 46.3]	39.7	[38.0, 43.8]	**0.7**	[−4.6, 10.6]	0.4
Body							
V_5Gy_ [cc]	2576.4	[1754.3, 3565.8]	2342.7	[1687.4, 2980.4]	**8.0**	[−30.1, 18.3]	**<0.001**
V_10Gy_ [cc]	871.9	[436.2, 1721.6]	938.8	[632.7, 1218.8]	**−13.6**	[−67.2, 29.7]	**0.04**
V_20Gy_ [cc]	207.1	[118.9, 359.7]	217.2	[138.4, 295.0]	**−6.5**	[−26.4, 18.0]	**0.01**
V_30Gy_ [cc]	110.6	[64.7, 176.4]	114.1	[73.6, 158.1]	**−4.2**	[−13.8, 10.3]	**0.01**
Plan Parameters							
MU	47377	[33598, 60794]	23524	[19873, 35512]	**48.7**	[6.9, 66.4]	**<0.001**
WSS [mm^2^]	451.0	[320.4, 660.0]	873.0	[560.6, 1248.5]	**−97.5**	[−161.2, −10.5]	**<0.001**
MCS	0.49	[0.31, 0.61]	0.50	[0.33, 0.60]	**−5.9**	[−51.3, 27.3]	0.2
NoF Beams	73.9	[51,115]	47.4	[37,59]	**26.5**	[6.0, 57.0]	**<0.001**
NoF Nodes	60.9	[46,90]	47.4	[37,59]	**13.4**	[−2.0, 42.0]	**<0.001**
NoF Segments	107.2	[54,235]	92.9	[54,167]	**8.9**	[−72.5, 62.1]	0.06
Estimated Delivery Time [min]	29.5	[20.1, 38.3]	21.5	[16.6, 31.6]	**26.7**	[−3.6, 42.5]	**<0.001**

^a^ percentage values are given as: (SO‐VOLO™)*100/SO.

^b^ Bold *P*‐values represent statistically significant results (*P* < 0.05).

#### 3.A.3 OAR and patient dose

**FIG. 2:**
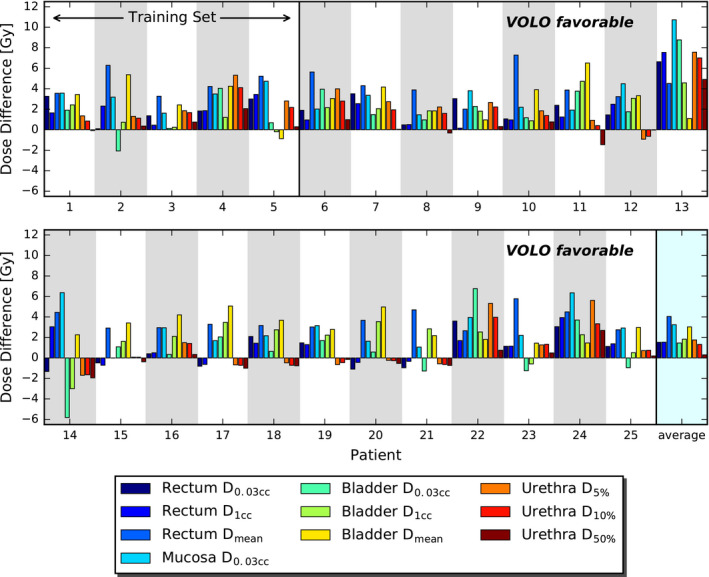
Comparison between SO and VOLO™ in OAR dose parameters for prostate SBRT. Bars present differences between SO and VOLO™ results. Positive values indicate that VOLO™ is favorable.

**FIG. 3:**
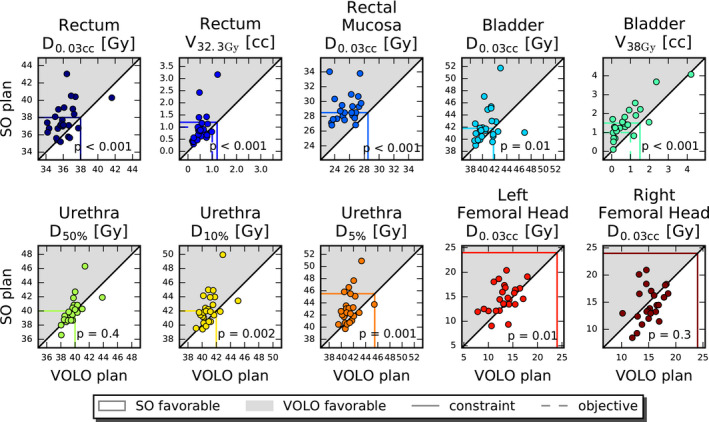
Mutual comparisons of SO and VOLO™ dosimetric prostate plan parameters. The horizontal and vertical colored lines show constraint (solid) and objective (dashed) values. Markers in the gray area point at superiority of VOLO™. Observed constraint violations (especially seen for SO) are due to the fixed PTV coverage of 95%.

**FIG. 4:**
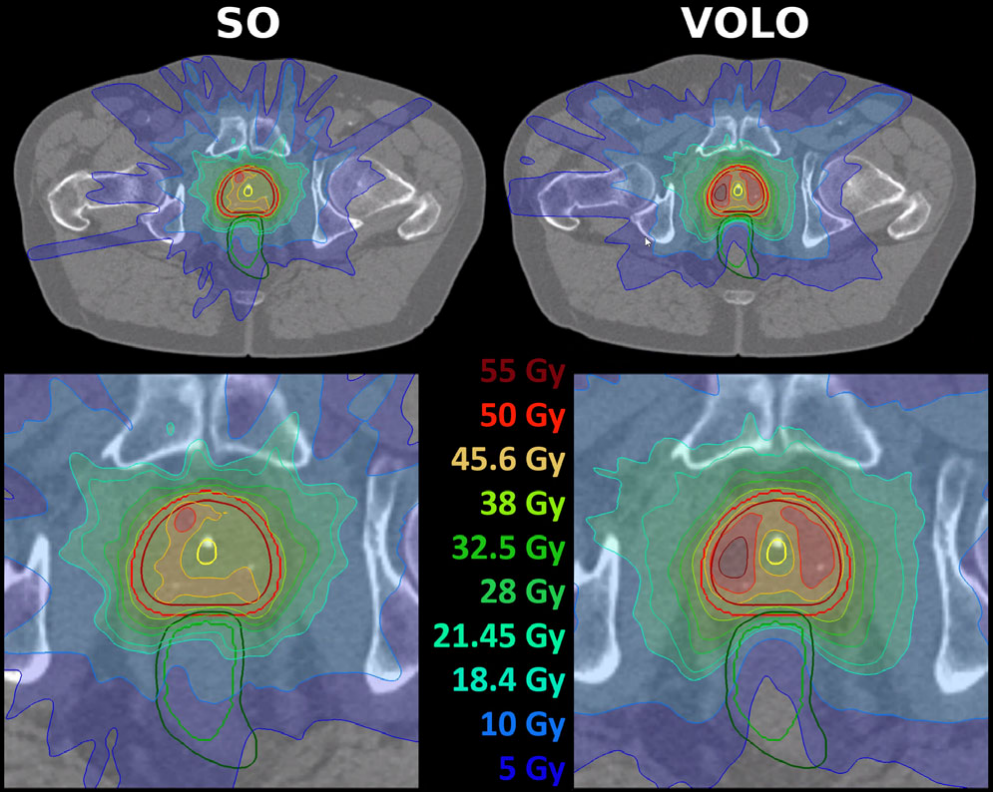
Upper panels: example of dose distributions (SO – left, VOLO™ ‐ right) for a prostate patient. Patient 19 was chosen as the dose difference between SO and VOLO™ in rectum D_0.03cc_ was for this patient closest to population average. Structures shown are: prostate and PTV – red, rectum – green and urethra – yellow. For both plans, the lower panel shows a zoomed area around the prostate.

For similar PTV dose, VOLO™ plans performed on average better than SO plans in almost all the studied parameters (Table 1). Rectum and bladder D_1cc_ were on average reduced by 4.7% with reductions up to 20.5% and 12.1%, respectively. Mean/maximum reduction in rectum and bladder D_mean_ were 34.3%/45.8% and 23.5%/41.0%, respectively. Also high urethra doses were reduced in the VOLO™ plans, with mean/maximum reductions of 3.8%/14.9% and 3.0%/14.0% in D_5%_ and D_10%_, respectively. The patient volume receiving 5 Gy or more was reduced by 8% (maximum 18.3%) in the VOLO™ plans with increases in volumes receiving higher doses, as visible in Table 1. Fig. 2 shows that the superiority of VOLO™ in OAR plan parameters was observed for all patients. In Fig. 3 dosimetric plan parameters are presented together with constraint and objective values, showing fewer and smaller violations for VOLO™. Dose distributions for a representative patient are shown in Fig. 4. Population mean DVHs are presented in Appendix S2, showing reduced spread of rectum and bladder doses, and urethra high doses with VOLO™.

#### 3.A.4 Nondosimetric plan parameters

**FIG. 5:**
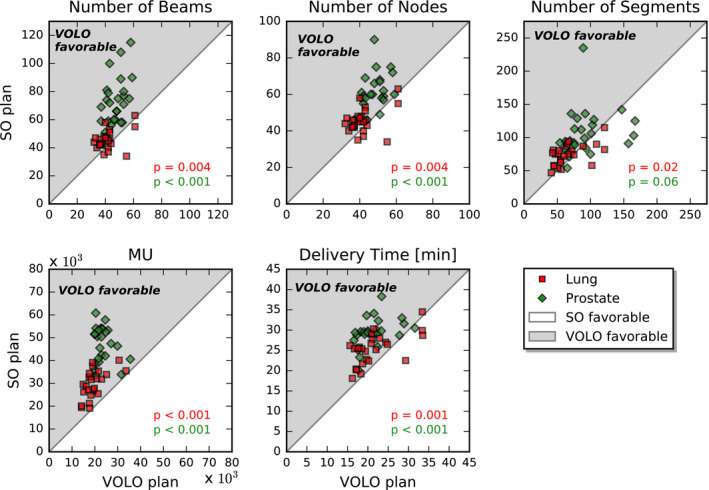
Comparison of nondosimetric plan parameters for prostate (green diamonds) and lung (red squares).

The VOLO™ prostate plans were highly favorable in terms of plan complexity and treatment time. The average WSS was significantly increased with VOLO™ as compared to SO (873.0 mm^2^ vs 451.0 mm^2^, *P* < 0.001). The MCS for VOLO™ was comparable to SO (0.49 vs 0.50, *P* = 0.2), showing similar complexity of SO and VOLO™ plans. With a comparable number of total MLC segments, mean/maximum reductions in MU were 48.7%/66.4%, and the number of beams and nodes were reduced by 26.5%/57.0% and 13.4%/42.0%, respectively (see Table 1 and Fig. 5). On average the estimated delivery time was reduced by 8 min (from 29.5 min to 21.5 min), with a maximum reduction of 15 min. All these reductions were statistically significant. Clinical SO planning took between 2 and 12 h (mean ~ 6 h), depending on the complexity of the case. For VOLO™ plans, the planning time was on average 1 h 10 min, with a range of 10 min to 3 h and 20 min.

### 3.B Lung

#### 3.B.1 Clinical acceptability of original VOLO™ plans

For the evaluating clinician (JN), all plans in the arbitrarily selected subset of 10 patients were clinically acceptable.

#### 3.B.2 PTV coverage and dose

PTV coverage achieved with SO ranged from 89.3% to 99.2%. Nine patients had SO PTV coverage below 98%; three between 89.3% and 97%, and six between 97% and 98%. Plans created with VOLO™ had a PTV coverage between 91.6% and 98.1%; of the three patients with a VOLO™ coverage below 98%, one patient had a coverage of 91.6%, and two between 97.5% and 98%. As mentioned in the Methods section, the aim was to generate for each patient a VOLO™ plan with a PTV D_max_ similar to the one in the SO plan. As shown in Table 2, a small difference of 1.9% in D_0.03cc_ was found, which was considered of minor clinical importance.

**TABLE 2:** Comparisons of plan parameters for lung cancer patients for plans generated with SO and VOLO™.

	SO		VOLO™		SO‐VOLO™ ^a^			
	Mean	Range	Mean	Range	Mean [%]	Range [%]	*P*‐value^b^
PTV							
D_98%_ [Gy]	55.0	[54.9, 55.1]	55.0	[54.9, 55.1]	**0.0**	[−0.2, 0.2]	0.3
D_0.03cc_ [Gy]	71.9	[67.3, 91.1]	73.2	[69.0, 89.6]	**‐1.9**	[−5.9, 1.7]	**0.001**
CI	1.2	[1.1, 1.8]	1.2	[1.1, 1.8]	**0.6**	[−14.4, 13.4]	0.5
Esophagus							
D_0.03cc_ [Gy]	15.4	[2.8, 35.0]	12.5	[1.5, 30.5]	**18.9**	[−4.4, 70.9]	**<0.001**
D_mean_ [Gy]	3.8	[0.7, 10.8]	3.3	[0.4, 9.4]	**13.4**	[−38.8, 62.8]	**0.002**
Main Bronchus							
D_0.03cc_ [Gy]	20.1	[0.5, 50.0]	18.1	[0.4, 44.7]	**11.1**	[−101.5, 57.6]	**0.003**
D_mean_ [Gy]	6.2	[0.2, 18.1]	5.8	[0.2, 15.1]	**7.6**	[−42.6, 50.4]	0.1
Skin							
D_0.03cc_ [Gy]	12.6	[6.6, 18.9]	12.5	[7.0, 18.3]	**‐1.9**	[−52.3, 24.5]	0.7
D_mean_ [Gy]	0.5	[0.2, 0.9]	0.4	[0.2, 0.9]	**2.8**	[−2.9, 8.4]	**0.002**
Spinal Cord							
D_0.03cc_ [Gy]	11.8	[1.1, 25.1]	10.4	[0.9, 22.7]	**13.2**	[−48.6, 65.9]	**0.01**
D_mean_ [Gy]	2.2	[0.4, 4.7]	2.0	[0.2, 4.4]	**11.2**	[−32.9, 70.1]	**0.02**
Thoracic Wall							
D_30cc_ [Gy]	18.1	[7.3, 35.9]	17.0	[8.3, 29.9]	**4.4**	[−13.7, 18.0]	**0.008**
D_mean_ [Gy]	4.8	[1.3, 14.0]	4.6	[1.4, 12.7]	**2.7**	[−12.5, 16.8]	**0.01**
Trachea							
D_0.03cc_ [Gy]	9.9	[0.3, 41.3]	8.5	[0.2, 37.3]	**16.1**	[−23.1, 45.5]	**0.001**
D_mean_ [Gy]	2.4	[0.1, 8.8]	2.2	[0.1, 9.9]	**11.9**	[−16.1, 47.0]	0.08
Lung							
V_5Gy_ [%]	18.8	[3.3, 37.5]	18.7	[3.6, 38.5]	**0.5**	[−22.4, 12.8]	0.6
V_16Gy_ [%]	5.6	[0.8, 11.4]	5.6	[0.8, 12.1]	**‐1.7**	[−19.8, 14.9]	0.5
V_20Gy_ [%]	4.1	[0.6, 8.3]	4.1	[0.6, 8.5]	**‐1.8**	[−21.0, 13.9]	0.6
V_30Gy_ [%]	2.3	[0.4, 4.7]	2.3	[0.4, 4.7]	**‐1.4**	[−20.5, 9.5]	0.6
Body							
V_5Gy_ [cc]	7.1	[2.0, 13.6]	6.9	[1.9, 13.5]	**3.8**	[−8.1, 19.2]	**0.006**
V_10Gy_ [cc]	2.8	[0.6, 7.3]	2.8	[0.8, 6.9]	**0.4**	[−16.9, 20.8]	0.1
V_20Gy_ [cc]	0.8	[0.2, 2.9]	0.8	[0.2, 2.8]	**2.5**	[−17.9, 20.8]	0.09
V_30Gy_ [cc]	0.4	[0.1, 1.4]	0.4	[0.1, 1.4]	**0.8**	[−15.9, 17.1]	0.5
Plan Parameters							
MU	29265	[19111, 40147]	19584	[14143, 33648]	**32.8**	[5.3, 51.4]	**<0.001**
WSS [mm^2^]	532.6	[273.8, 1198.1]	864.3	[349.0, 2096.6]	**‐58.4**	[−116.2, −7.4]	**<0.001**
MCS	0.43	[0.33, 0.53]	0.53	[0.45, 0.65]	**‐24.6**	[−74.4, 10.8]	**<0.001**
NoF Beams	45.5	[34,63]	41.3	[32,61]	**7.9**	[−61.8, 31.0]	**0.003**
NoF Nodes	45.5	[34,63]	41.3	[32,61]	**7.9**	[−61.8, 31.0]	**0.003**
NoF Segments	74.3	[47,115]	66.1	[41,121]	**10.5**	[−75.9, 45.7]	**0.02**
Estimated Delivery Time [min]	25.4	[18.1, 34.5]	21.6	[15.6, 33.5]	**14.5**	[−30.2, 40.5]	**<0.001**

^a^ percentage values are given as: (SO‐VOLO™)*100/SO.

^b^ Bold *P*‐values represent statistically significant results (*P* < 0.05).

For patient 23 with the lowest clinically achieved PTV coverage (89.3%), the constraint for stomach D_max_ was limiting. With VOLO™, it was possible to increase the coverage to 91.6% while keeping the stomach dose within the constraint. For this patient, there was a higher bowel dose observed for VOLO™, which was however far from constraint. Patient 23 was the only patient with stomach and bowel structures involved. Therefore, no statistical analyses could be performed for these OARs. Patient 14 had a tumor located close to spinal cord. A PRV (spinal cord + 5 mm margin) was therefore used for planning of this patient instead of the spinal cord itself. Related to this, a PTV coverage of 95.9% was accepted for the clinical SO plan. With VOLO™ it was possible to reduce the spinal cord maximum PRV dose by 4.4 Gy, while keeping 98% PTV coverage. The main bronchus was in SO planning a limiting structure for patient 25, the achieved PTV coverage was 95.3%. With VOLO™ it was possible to increase the coverage to 97.5%, while still keeping the main bronchus D_max_ within constraint. Tradeoffs for all lung patients are presented in electronic Appendix S3.

Population mean PTV DVHs after renormalization to the preferred coverage of 98% for each patient are presented in Fig. 1(b), showing large similarity for SO and VOLO™.

#### 3.B.3 OAR and patient doses

**FIG. 6:**
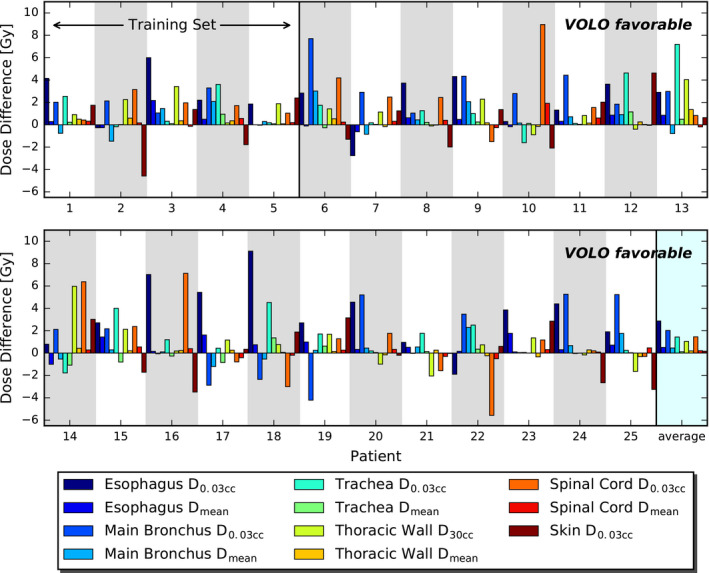
Comparisons between SO and VOLO™ OAR dose parameters for all lung cancer patients separately. Bars present differences between SO and VOLO™ results; positive value indicates that VOLO™ is favorable.

**FIG. 7:**
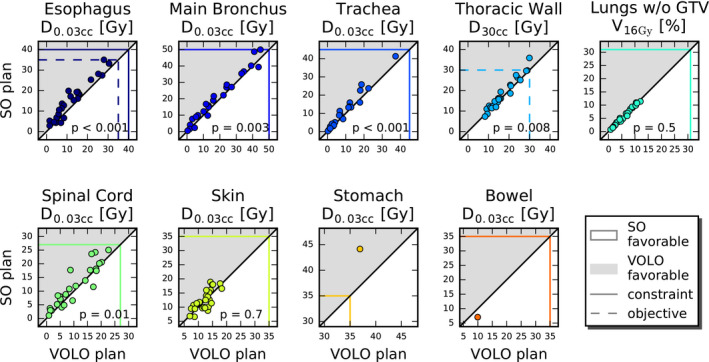
Mutual comparisons of SO and VOLO™ dosimetric lung plan parameters. The horizontal and vertical colored lines show constraint (solid) and objective (dashed) values. Markers in the gray area point at superiority of VOLO™. Observed constraint violations (especially seen for SO) are due to the fixed PTV coverage of 98%.

**FIG. 8:**
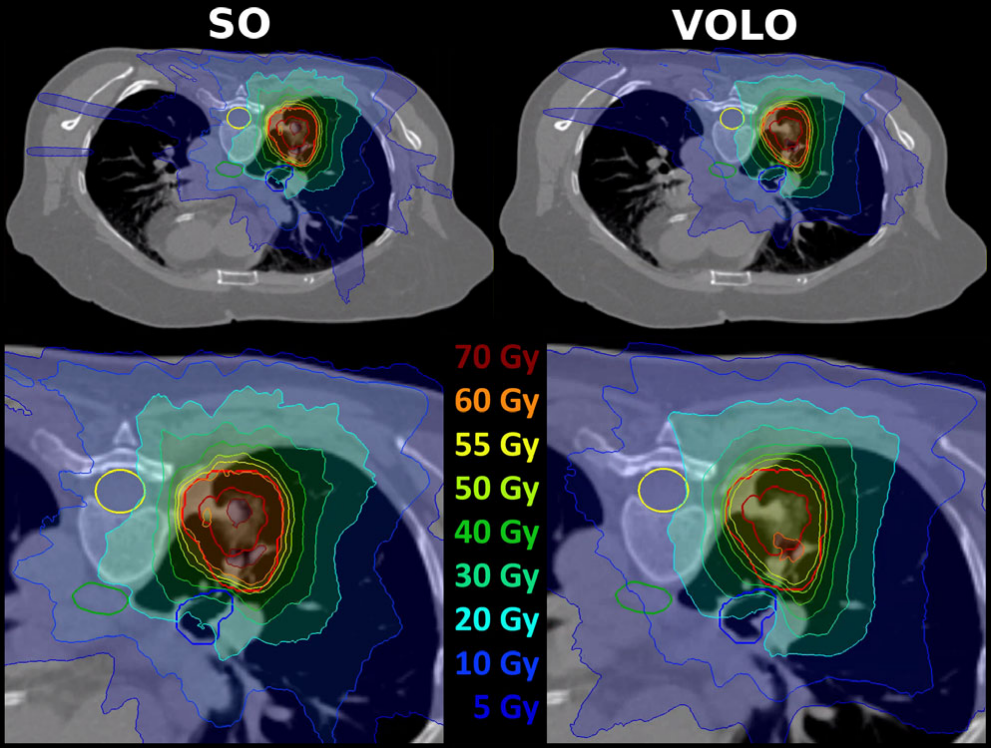
Upper panels: example of dose distributions (SO – left, VOLO™ ‐ right) for lung patient. Patient 1 was chosen as the dose difference between SO and VOLO™ in main bronchus D_0.03cc_ was for this patient closest to population average. Structures shown are: CTV and PTV – red, esophagus ‐ green, spinal cord ‐ yellow and main bronchus – blue. For both plans, the lower panel shows a zoomed area around the target volume.

After renormalization of all SO and VOLO™ plans to a PTV coverage of 98%, VOLO™ plans performed on average better than SO plans in most healthy tissues (Table 2), with large variations among patients (Fig. 6). Esophagus, main bronchus, spinal cord, and trachea D_0.03cc_ were on average reduced by 18.9%, 11.1%, 13.2%, and 16.1% with maximum reductions up to 70.9%, 57.6%, 65.9%, and 45.5%, respectively. Mean/maximum reduction in esophagus, spinal cord and thoracic wall D_mean_ were 13.4%/62.8%, 11.2%/70.1% and 2.7%/16.8%, respectively. Also, thoracic wall D_30cc_ was reduced in VOLO™ plans, with mean/maximum reductions of 4.4%/18.0%. Lung dose was comparable. The patient volume receiving 5 Gy or more was reduced by 3.8% (maximum 19.2%) in the VOLO™ plans with similar volumes receiving higher doses. In Fig. 7 dosimetric plan parameters are presented together with constraint and objective values. Dose distributions for a representative patient are shown in Fig. 8. Appendix S4 shows population mean DVHs for the investigated planning approaches.

#### 3.B.4 Nondosimetric plan parameters

The VOLO™ lung plans were favorable in terms of plan complexity and treatment time. The average WSS was significantly increased with VOLO™ as compared to SO (864.3 mm^2^ vs 532.6 mm^2^, *P* < 0.001). The MCS for VOLO™ was on average higher compared to SO (0.53 vs. 0.43, *P* < 0.001), showing smaller average complexity of VOLO™ plans. Mean/maximum reductions in MU were 32.8%/51.4%, and the number of beams, nodes and segments was reduced by 7.9%/31.0% and 7.9%/31.0% and 10.5%/45.7%, respectively (see Table 2 and Fig. 5). On average, the estimated delivery time was reduced by 3.7 min (from 25.4 min to 21.6 min), with a maximum reduction of 10 min. All these reductions were statistically significant. Clinical SO planning took between 1 and 8 h (mean ~ 3 h), depending on the complexity of the case. For VOLO™, the planning time was on average 1 h 40 min, with a range of 30 min to 6 h.

## 4 DISCUSSION

The main goal of this study was to validate the new VOLO™ optimizer for treatment planning for MLC‐based robotic SBRT for prostate cancer with intended urethra sparing, and for central lung tumors with many OARs nearby. As summarized in the Methods section, VOLO™ has a rather different inverse planning approach compared to the existing SO, it uses a quasi‐Newton optimizer with fast convergence^14^ and it operates with faster computer hardware (GPU vs. CPU). For 25 prostate and 25 lung cancer patients, previously treated with robotic SBRT with an SO plan, VOLO™ was used for replanning for the same planning aims as used for the clinical SO planning. For prostate cancer, the VOLO™ plans were dosimetrically highly superior compared to the clinically delivered plans, which held for all OARs and patients. For lung cancer there was also an overall dosimetric gain with VOLO™, but it was more dependent on OAR and patient. For both treatment sites there were large gains in number of MU and number of beams and nodes, with clinically relevant reductions in treatment delivery times. Observed reductions in planning time were also large. In our opinion, the observed enhanced OAR sparing with VOLO™ is clinically meaningful and the reduced planning and treatment times are highly relevant from the logistical point of view.

In principle, plan quality differences between SO and VOLO™ can originate from differences in beam segments/intensity profiles and differences in selected beam directions, depending on nodes and isocenter placement. In this study, for both prostate and lung, planning with SO and VOLO™ started with the full set of available nodes (Methods section), with some blocked because of applied blocking structures. For prostate cancer, there were substantial differences in both: the applied numbers of nodes, numbers of MLC segments, WSS and total MU for SO/VOLO™ were 60.9/47.4, 107.2/92.9, 451.0/873.0, 47377/23524. Of the on average 47.4 nodes in VOLO™ plans, only 67% were also present in the SO beam set. Clearly, these differences in beam directions could have contributed to the observed differences in plan quality. On the other hand, also the numbers of segments, WSS and total MU were rather different, pointing at differences in intensity profiles. For lung, total numbers of nodes were rather similar: 45.5 for SO and 41.3 for VOLO™. However, on average only 65.2% of directions used in SO were also used in VOLO™. So effectively, also for lung there are substantial differences in selected beam directions. However, also for lung, differences in number of segments, WSS and total MU were significant: 74.3/66.1, 532.6/864.3, 29265/19584 (SO/VOLO™) pointing at differences in intensity profiles.

Schüler et al.^1^ also compared VOLO™ with SO. They investigated six groups of five patients: simple brain treated with the IRIS™ variable aperture collimator, complex brain treated with either the IRIS™ or the InCise™ MLC, complex spine treated with the IRIS™ or the InCise™ MLC, and prostate treated with the InCise™ MLC. SO planning was used for treatment. For all patients, an alternative plan was made with VOLO™, aiming to meet OAR constraints in the SO plans and improve on MU and delivery time. In line with our study, they also found reductions in MU and delivery time, which were a bit smaller than we observed, i.e., −38% vs. −49% and −17% vs. −27%, respectively. For the five prostate patients, they found only minor differences in dosimetric plan quality. This is in contrast with the results for the 25 prostate patients in our study; not only did we observe drastically reduced MU and delivery times, but there were also large dose reductions in healthy tissues (Table 1). This difference in OAR doses could possibly (partially) be explained by a difference in study design. While they used VOLO™ to reduce the MU and delivery times for OAR doses similar to those obtained with SO (above), we actively tried to reduce OAR doses as much as possible (not knowing about obtained doses in the SO plans). Apparently, VOLO™ planning by Schüler et al. did not result in better dosimetric plan quality if not explicitly desired, or better quality could possibly only be obtained with smaller gains in MU and delivery times. An alternative (partial) explanation for the differences between the studies in OAR doses could be in the planning aims for the urethra. As mentioned above, in our study the urethra dose was actively restricted which resulted in a dose valley in and around the urethra. This approach was not applied by Schüler et al. Probably, the upfront geometrical segment generation in SO planning (Methods section, 2.B.1.) was less suited for the inhomogeneous dose distributions required in our center, resulting in relatively large dosimetric gains for VOLO™ in which segments are dosimetrically optimized.

Zeverino et al.^3^ compared SO and VOLO™ for brain, spine, prostate, and lung cancer patients, treated with the circular IRIS™ collimator (10 patients per site). Another 10 brain patients were planned for the InCise™ MLC to a different prescription dose. For prostate and lung there were no MLC plans generated, complicating comparisons with our study with MLC treatment only. In line with the work by Schüler et al^1^ and our study, they also observed reductions in number of nodes (36%), number of beams (14%) and MU (31%). For prostate patients, significant dose reductions were only reported for the urethra, with averages of 66.7%, 75.0% and 5.2% in V_39Gy_, V_41Gy_ and D_max_, respectively. In our study, enhanced OAR sparing with VOLO™ planning was largest for rectum and bladder and less for the urethra. It is not clear to what extent this could be caused by differences in the clinical planning aims or the use of different collimators, i.e., the InCise™ MLC in our study and the circular IRIS™ by Zeverino et al. For lung patients, Zeverino et al. did not find differences in OAR doses achieved with SO and VOLO™, while in our study large improvement was observed with VOLO™ planning, depending on OAR and patient (Table 2). Possibly, this is due to patient selection. In our study we included central lung tumors, while patient type was not specified by Zeverino et al. Possibly, also the different collimation in the two centers contributes to these differences.

Calusi et al.^2^ compared VOLO™ with SO for MLC‐based SBRT for liver, prostate, pancreas, and spine. In total 25 patients were included in their study, five to seven per location. The authors found reductions in MU/prescribed dose, delivery time, number of nodes, and number of segments of 19%, 15%, 12% and 23%, respectively. However no significant differences in OAR doses were reported.

The three published studies^1^, ^2^, ^3^ and our study all show large improvements in treatment efficiency (MU, numbers of beams, delivery times) with VOLO™ planning. On the other hand, enhanced OAR sparing was only seen by Zeverino et al.^3^ and in our study. In both studies improvements were seen for prostate cancer, but Zeverino et al. only observed urethra sparing while in our study there was also important sparing of rectum and bladder. Significant OAR sparing in lung cancer treatment was only observed in our study. Study design, case complexity and applied collimator seem to impact the observations for OAR.

It was previously shown^19^, ^20^ that knowledge‐based planning (using planning CT‐scans and plans from previously treated patients) for predicting feasible constraints for use in SO planning for robotic prostate SBRT could reduce dose in rectum and bladder. Recent studies^21^, ^22^ investigated SO planning based on patient‐specific constraints and objectives values, obtained from a preoptimization with a system for automated multi‐criterial plan generation. It was observed that with the preoptimized constraints and objectives, the quality of the SO plans significantly improved compared to SO planning in clinical practice without the input of the preoptimizer. The latter observations suggest that generating high‐quality plans with SO can in practice be hindered by problems in finding optimal patient‐specific constraints and objective values in the clinical interactive trial‐and‐error planning.

Interestingly, the enhanced OAR sparing in the VOLO™ plans as observed in this study was obtained by a planner with no previous experience in SBRT or CK planning, with planning times that were often much reduced compared to clinical planning. Also here we attribute the inferior quality of the SO plans at least in part to difficulties for the clinical planners in steering the SO algorithm to the best possible plans, i.e., finding optimal patient‐specific goal values for the cost functions in the trial‐and‐error planning effort. In general, our experience was that the trial‐and‐error planning was less intuitive for SO than for the VOLO™ algorithm, making it harder to find good constraints defining cost functions. Due to the enhanced calculation speed of the VOLO™ algorithm, more iterations could be made for finding the appropriate cost functions. Influence of calculation speed on plan quality was previously observed.^23^ The difference between SO and VOLO™ in calculation speed could be related to the difference in the applied computer hardware (GPU vs. CPU) and improved optimizer convergence which is used without hard constraints. Additionally, the SO algorithm uses a higher dose threshold when storing the per‐beam dose maps that are used during optimization, which can cause larger differences between the optimization result and the final dose calculation, possibly requiring additional optimization iterations for good solutions, enhancing the total planning time. In this paper we compared the combination (unexperienced planner/VOLO™) with (experienced planner/SO). The technical advantages of VOLO™ compared to SO (above) allowed the unexperienced planner to beat the experienced planner in overall plan quality. Possibly, with the combination (experienced planner/VOLO™), plan quality could have been further enhanced. At the time of the study, plan generation with this combination was not feasible. On the other hand, this additional planning work would not have changed the main conclusion of the paper: VOLO™ has the potential of increasing plan quality compared to SO. The extra combination could only have further strengthened this conclusion.

As mentioned above, another major difference between SO and VOLO™ was the generation of the MLC segments. With SO, segment shapes are preselected based on beam’s‐eye‐view projections, while in the VOLO™ algorithm, segment shapes are dosimetrically optimized with the usage of objective functions used also in the fluence optimization step. This difference may in part also be responsible for the observed overall higher quality of the VOLO™ plans, especially for prostate cancer with the prescribed inhomogeneous dose distributions with urethra sparing. This enhanced dosimetric quality could be obtained with large reductions in MU, numbers of beams and numbers of nodes, while the WSS was clearly increased pointing at improved plan robustness. MCS for prostate was comparable for SO and VOLO™ while being reduced with VOLO™ for lung.

As demonstrated in this study, the applied inverse planning algorithm can have a major impact on obtained plan quality. This needs to be considered in treatment planning studies for treatment technique comparisons; observed differences in plan quality can be due to differences in techniques, but differences in applied optimizers can heavily bias the results especially if optimization is done manually. Even more important, this study points at the need of continued research on optimization algorithms, and their use for benchmarking clinical algorithms to avoid patient treatment on advanced machines with suboptimal plans.

## 5 Conclusions

In robotic SBRT with the InCise™ 2 MLC, the novel VOLO™ inverse planning algorithm was highly superior compared to planning with Sequential Optimization (SO) for two complex patient groups, i.e., prostate cancer treated with urethra sparing, and central lung tumors. Apart from large dosimetric advantages, also MU, numbers of beams, numbers of nodes, treatment delivery times, and planning times substantially improved. Treatment times reduced by 8 min and 3.8 min for prostate and lung, respectively. This study points out that more comparative studies on optimizers are needed. Such studies may raise awareness among users and TPS vendors of potential weaknesses, and may avoid suboptimal treatment on high‐end treatment units, and erroneous conclusions from treatment planning studies.

## Conflict of interest

This work was in part funded by a research grant of Accuray Inc. Erasmus MC Cancer Institute also has research collaborations with Elekta AB Stockholm, Sweden. No additional external funding was received for this study. The funders had no role in study design, data collection and analysis, and decisions on preparation of the manuscript and publication.

## Author Contribution

Marta K. Giżyńska: study design and concept, data collection and analysis, manuscript writing. Linda Rossi: study design and concept, manuscript revision. Wilhelm den Toom: data collection and interpretation, manuscript revision. Maaike T. W. Milder: data interpretation, manuscript revision. Kim C. de Vries: data analysis, manuscript revision. Joost J. Nuyttens: data analysis, manuscript revision. Ben J. M. Heijmen: study design and concept, manuscript revision.

## Supporting information


**Appendix S1.** Tradeoffs of all parameters for prostate plans. Color represents the percentage difference between SO and VOLO™ plans calculated as (SO‐VOLO™)*100/SO.

**Appendix S2.** Population mean DVHs for bladder, rectum and urethra for planning with SO and with VOLO™.

**Appendix S3.** Tradeoffs of all parameters for lung plans. Color represents the percentage difference between SO and VOLO™ plans calculated as (SO‐VOLO™)*100/SO.

**Appendix S4.** Population mean DVHs OARs as defined for lung planning with SO and with VOLO™.
