# Supplementary material for: Largely reduced OAR doses, and planning and delivery times for challenging robotic SBRT cases, obtained with a novel optimizer
Source: J Appl Clin Med Phys. 2021 Jan 21;22(3):35–47. doi: 10.1002/acm2.13172 (PMC7984474; doi:10.1002/acm2.13172)

Appendix S1

Tradeoffs of all parameters for prostate plans. Color represents the percentage difference between SO and VOLO™ plans calculated as (SO-VOLO™)*100/SO.

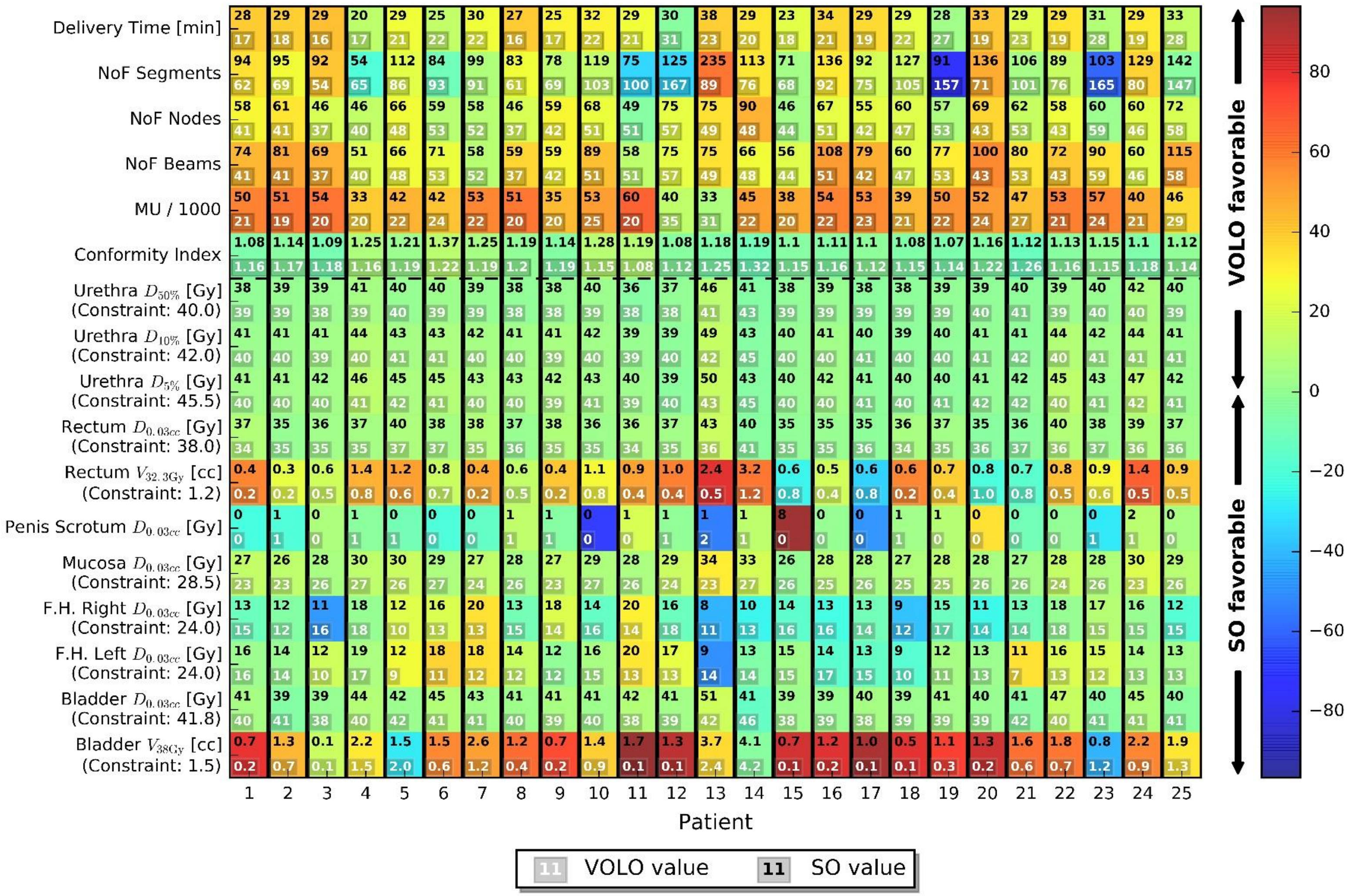

Supplement: Supplementary file 1 — Appendix S1. Tradeoffs of all parameters for prostate plans. Color represents the percentage difference between SO and VOLO™ plans calculated as (SO‐VOLO™)*100/SO. [file ACM2-22-35-s004.pdf]
