# Supplementary figures and images for: Largely reduced OAR doses, and planning and delivery times for challenging robotic SBRT cases, obtained with a novel optimizer
Source: J Appl Clin Med Phys. 2021 Jan 21;22(3):35–47. doi: 10.1002/acm2.13172 (PMC7984474; doi:10.1002/acm2.13172)

## Appendix S4

Population mean DVHs OARs as defined for lung planning with SO and with VOLO™.

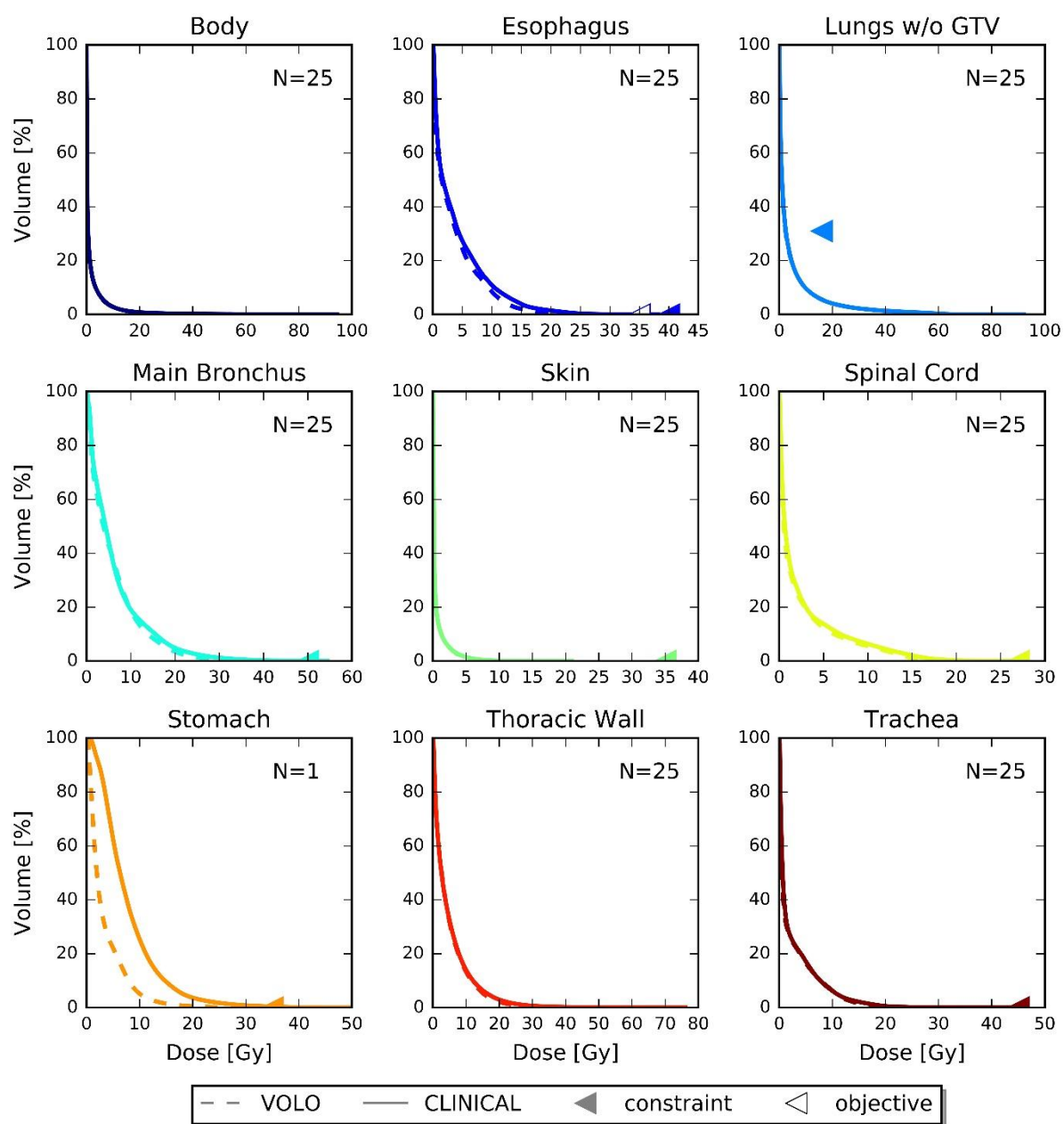

Supplement: Supplementary file 4 — Appendix S4. Population mean DVHs OARs as defined for lung planning with SO and with VOLO™. [file ACM2-22-35-s001.pdf]
